# Attentional reorienting triggers spatial asymmetries in a search task with cross-modal spatial cueing

**DOI:** 10.1371/journal.pone.0190677

**Published:** 2018-01-02

**Authors:** Rebecca E. Paladini, Lorenzo Diana, Giuseppe A. Zito, Thomas Nyffeler, Patric Wyss, Urs P. Mosimann, René M. Müri, Tobias Nef, Dario Cazzoli

**Affiliations:** 1 Gerontechnology and Rehabilitation Group, University of Bern, Bern, Switzerland; 2 Perception and Eye Movement Laboratory, Departments of Neurology and Clinical Research, University Hospital Inselspital and University of Bern, Bern, Switzerland; 3 Department of Bioengineering, Imperial College London, London, United Kingdom; 4 Center of Neurology and Neurorehabilitation, Luzerner Kantonsspital, Switzerland; 5 University Hospital of Old Age Psychiatry, University of Bern, Bern, Switzerland; 6 ARTORG Center for Biomedical Engineering Research, University of Bern, Bern, Switzerland; Tsinghua University, CHINA

## Abstract

Cross-modal spatial cueing can affect performance in a visual search task. For example, search performance improves if a visual target and an auditory cue originate from the same spatial location, and it deteriorates if they originate from different locations. Moreover, it has recently been postulated that multisensory settings, i.e., experimental settings, in which critical stimuli are concurrently presented in different sensory modalities (e.g., visual and auditory), may trigger asymmetries in visuospatial attention. Thereby, a facilitation has been observed for visual stimuli presented in the right compared to the left visual space. However, it remains unclear whether auditory cueing of attention differentially affects search performance in the left and the right hemifields in audio-visual search tasks. The present study investigated whether spatial asymmetries would occur in a search task with cross-modal spatial cueing. Participants completed a visual search task that contained no auditory cues (i.e., unimodal visual condition), spatially congruent, spatially incongruent, and spatially non-informative auditory cues. To further assess participants’ accuracy in localising the auditory cues, a unimodal auditory spatial localisation task was also administered. The results demonstrated no left/right asymmetries in the unimodal visual search condition. Both an additional incongruent, as well as a spatially non-informative, auditory cue resulted in lateral asymmetries. Thereby, search times were increased for targets presented in the left compared to the right hemifield. No such spatial asymmetry was observed in the congruent condition. However, participants’ performance in the congruent condition was modulated by their tone localisation accuracy. The findings of the present study demonstrate that spatial asymmetries in multisensory processing depend on the validity of the cross-modal cues, and occur under specific attentional conditions, i.e., when visual attention has to be reoriented towards the left hemifield.

## Introduction

In everyday life, we perceive our surroundings by integrating information obtained from all of our senses. For instance, when searching for a mislaid object, such as a cell phone, or when looking for potential sources of danger on a busy street, we do not solely rely on our vision, but also on our sense of hearing. Indeed, previous research has shown that the presentation of auditory stimuli can significantly influence participants’ performance during visual target detection (e.g., [1, 2]). More precisely, cross-modal spatial cueing paradigms have shown that spatial congruency between an auditory cue and a visual target can significantly decrease participants’ reaction times, as compared to visual search tasks with spatially non-informative auditory cues [3–5], visual search tasks without any auditory stimuli [6, 7], or target detection tasks with spatially incongruent auditory cues (e.g., [8]).

In a search task with cross-modal spatial cueing, the beneficial effect of the spatial congruency between an auditory stimulus and a visual target has been commonly explained by means of attentional factors. In general, visual search tasks encompass the deployment of attention in space. According to Posner and Petersen [9], when deployed in space, attention has first to be disengaged from the current object, and then shifted and reengaged onto a new object. Thereby, in an overt visual search task (i.e., when the eyes are allowed to move freely), eyes and attention typically shift together in space [10, 11]. Thus, in a unimodal, overt visual search task requiring serial search (i.e., when attention is sequentially focused on each item on the display [12]), gaze and visual attention are continuously shifted across the entire visual search field, until the target is found. If, however, a spatially congruent auditory cue is presented, visual attention is oriented to the perceived origin of the cue, aligning the fovea with the latter [3]. Thus, the spatial auditory cue limits the effective extent of the visual search field, and enhances visual attentional processing in the cued area, which results in an improved target detection performance [13, 14]. If, however, the spatial origin of the auditory cue and the position of the visual target do not coincide, attention has to first be disengaged from the incorrectly cued location and shifted (i.e., reoriented) to the target location, which results in an impaired target detection performance (e.g., [2, 13, 15]).

It was recently proposed that multisensory settings, i.e., experimental settings, in which critical stimuli are concurrently presented in different sensory modalities (e.g., visual and auditory), might lead to spatial asymmetries in visual processing, ultimately resulting in beneficial effects for stimuli presented in the right hemifield [16]. For instance, a facilitation of visual identification in the right hemifield has been observed in a multisensory, audio-visual processing task ([17]; data reanalysed in [16]). This asymmetry was attributed to either a rightward bias of cross-modal attention, or to a rightward bias of attention per se (i.e., not just cross-modal attention), particularly in tasks with high perceptual load [16, 18, 19]. If the spatial asymmetries in visual processing within audio-visual multisensory settings are the results of attentional biases, then one would expect to observe asymmetrical effects also in paradigms in which visual attention is cued cross-modally, such as in audio-visual spatial cueing tasks. However, to the best of our knowledge, this hypothesis has not been investigated so far.

The aim of the present study consisted in investigating whether spatial attentional asymmetries would occur in an overt search task with cross-modal spatial cueing. Healthy participants completed a cross-modal search task with high perceptual load, in several different search conditions: a purely visual search condition (which served as a baseline, to be compared with various cross-modal conditions), a congruent cross-modal condition (in which the spatial origin of the auditory cue and the visual target coincided), an incongruent condition (in which the auditory cue and the visual target were localised in opposite hemifields), and a mono sound condition (in which the auditory cue did not contain any spatial information). The implementation of these different cross-modal conditions enabled us to assess under which specific circumstances spatial asymmetries in visual attention occur within a cross-modal cueing task. Moreover, to ensure that participants could localise the origin of the spatial auditory cues, and to investigate whether any asymmetries would occur in a purely auditory condition, participants also completed an auditory spatial localisation task.

## Materials and methods

### Participants

A total of 57 healthy volunteers, with a mean age of 24 years (standard deviation (SD) = 7, range = 18–58), participated in the study. 30 were women and 53 were right-handed. All participants had normal or corrected-to-normal vision and no history of hearing problems. They all gave written informed consent prior to the beginning of the experiment. The study was approved by the Ethics Committee of the State of Bern, and was conducted in compliance with the latest version of the Declaration of Helsinki.

### Stimuli

#### Auditory spatial localisation task

For this task, the visual display (i.e., a large touchscreen, see Apparatus section) was divided into 16 areas of equal size (i.e., 30.25 x 17 cm/ 480 x 270 pixels), according to an imaginary 4x4 grid. Participants were presented with an auditory stimulus (i.e., a 500 Hz sinusoidal tone) that could originate from the centre of one of these 16 areas. Participants were instructed to locate the origin of the tone as precisely as possible, by touching the corresponding point on the screen with a stick. The auditory spatial localisation task was composed of 32 trials (i.e., each position was tested twice). Each trial started with a black central fixation cross on a white background, presented for 1.5 s. Afterwards, the tone was reproduced for 2 s, and its origin had to be localised on a black blank screen.

#### Search task with cross-modal spatial cueing

**Fig 1:**
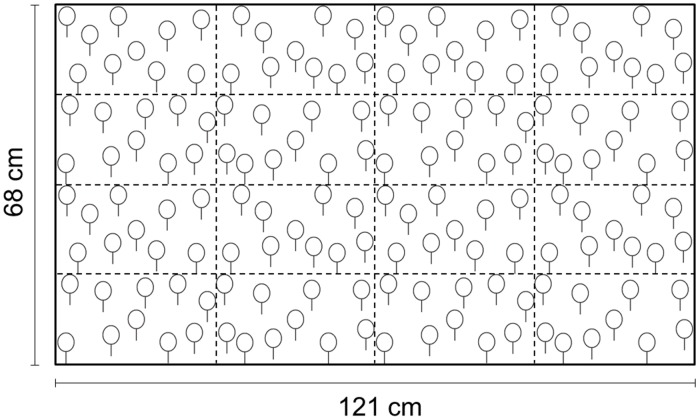
Example of a visual search array. The visual search array was subdivided into 16 possible target areas (dashed lines). The dashed lines are depicted for illustration purposes only (i.e., were not visible to the participants). In this example, the target (i.e., the only balloon not connected to a string) is located in the upper left area.

The task was based on an adapted and computerised version of the Balloon Test ([20]; see Fig 1). Participants were instructed to search an array of stylised balloons (circles with adjacent vertical lines, representing the string) presented on the touchscreen, in order to locate a single balloon that was not connected to a string (i.e., a simple circle). This balloon was the designated target, whereas the other balloons with strings represented distractors. The target balloon was embedded amongst 159 distractors, and it could appear at the centre of 16 possible areas determined according to an imaginary 4x4 grid (i.e., an identical division of the screen as in the auditory spatial localisation task; see Fig 1).

**Fig 2:**
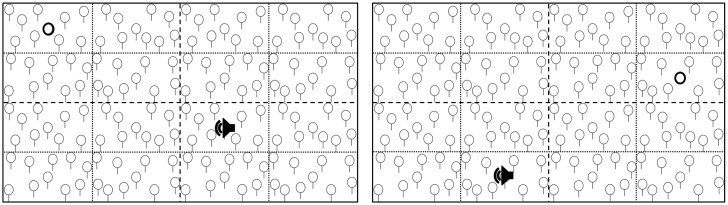
Examples of incongruent trials. Dotted lines delimit target areas, and dashed lines delimit quadrants. The bold circles represent the targets, and the black loudspeaker symbols represent the origin of the auditory cue. Target areas, quadrant, target positions, and cue origins are highlighted for illustration purposes only (i.e., were not visible to the participants).

In some trials (see below), auditory stimuli were presented through stereo headphones at the beginning of the visual search task (i.e., at a stimulus onset asynchrony, SOA, of 0 ms). The auditory stimuli were identical to the ones presented in the auditory spatial localisation task (i.e., 500 Hz sinusoidal tones), and they spatially originated from the centre of the same 16 possible areas that could contain the visual target. The task included 4 conditions of target-tone spatial congruency: a congruent condition (i.e., spatial origin of the tone and target location coincided); an incongruent condition (i.e., spatial origin of the tone and target location did not coincide); a mono sound condition (i.e., a binaural tone with no spatial properties was presented); and a no sound condition (i.e., no tone was presented). In the incongruent trials, the origin of the tone was localised on the other side of the horizontal as well as the vertical meridian (i.e., the spatial disparity between the visual target and the auditory cue equalled 59.94°). Thus, when dividing the screen into 4 equal quadrants (as depicted in Fig 2), the tone appeared in the diagonally adjacent quadrant with respect to the target, in the same target area within the quadrant.

Overall, the task included 128 trials. Specifically, the target appeared in each of the 16 possible target areas 8 times, with the following ratio between conditions of target-tone spatial congruency: 3 times in the congruent condition, once in the incongruent condition, twice in the mono sound condition, and twice in the no sound condition. This resulted in a 3 to 1 ratio between congruent and incongruent trials, which was meant to encourage participants to exploit the tone as a cue for their visual search. A more balanced ratio might have reduced or eliminated any effect of spatial congruence/incongruence between target and sound [13].

Each trial started with a black fixation cross, presented at the screen centre against a white background for 1.5 s, and was followed by the visual search array. As soon as the visual search array appeared, where applicable, the auditory cue was presented for 2s. The search display remained on screen until the target was found and touched.

### Apparatus

The experimental setting and the material used for the auditory spatial localisation task and the search task with cross-modal spatial cueing were identical. The experiment was carried out in a dimly lit room. Participants were seated at a distance of 60 cm from a 54.6” (121 x 68 cm/1920 x 1080 pixels) touchscreen monitor (5501LT, Elo Touch Solutions, Inc., Milpitas, CA), with their head stabilised by means of a chin-and-head rest. Participants were provided with a 60 cm-long stick, in order to be able to easily reach every position of the touchscreen.

Tones were presented by means of stereo headphones (E50 BT, JBL, Harman International Industries, Inc., Stamford, CT). To obtain auditory stimuli with different spatial coordinates, we adopted a binaural recording approach, a procedure known to accurately reproduce human auditory perception [21]. More precisely, a dummy head (B1-E, Binaural Enthusiast, Pisarzowice, Poland), with binaural stereo microphones placed in the ear canals, was positioned at 60 cm from the screen and stabilised on the chin-and-head rest. Subsequently, a loudspeaker was placed at the centre of each of the 16 aforementioned target areas, and a 500 Hz sinusoidal tone was reproduced for 2 s and recorded by means of the dummy head binaural microphones. The 16 auditory spatial stimuli were obtained with this procedure, and were used for the congruent and incongruent trials. For the mono sound trials, a 500 Hz sinusoidal tone, without any specific spatial information, was presented binaurally, i.e., an equal amount of auditory information was presented to the left and the right ear. The decibel level at which the auditory stimuli were presented was adjusted individually for each participant at the beginning of the experiment, in order to be clearly audible but not unpleasant. The individual decibel level for each participant was recorded, and it ranged from 60 to 75 dB, which is within the range used by previous studies (see e.g., [2, 3, 14, 22]).

Visual search stimuli and auditory stimuli were implemented in the computerised task by means of Unity 3D (Unity 5, Unity Technologies, San Francisco, CA).

### Procedure

#### Auditory spatial localisation task

The auditory spatial localisation task included 32 trials, i.e., the tone was presented twice at each possible spatial location, in a randomised order.

#### Search task with cross-modal spatial cueing

Before starting the search task with cross-modal spatial cueing, participants completed a short practice block, in order to familiarise themselves with the different conditions of target-tone spatial congruency. The practice block included 4 trials, i.e., each target-tone spatial congruency condition was presented once. The 128 trials of the search task with cross-modal spatial cueing were then administered, subdivided into 4 blocks of 32 trials each. The order of the trials was randomised. Between each block, participants were allowed to take a break of 2 minutes. Participants were instructed to look for the visual target by moving their eyes and to refrain from moving their head.

### Data analyses

To investigate the potential asymmetrical effects of cross-modal spatial cueing on search time in the visual search task, the touchscreen was subdivided into a left and a right side (60.5 cm/960 pixels each), both for the analysis of the results of the auditory spatial localisation task and of the search task with cross-modal spatial cueing.

Subsequently, in order to perform more fine-grained analyses of the data of both tasks, we further subdivided the touchscreen into four vertical columns (left peripheral, left central, right central, right peripheral; 30.25 cm/480 pixels each).

#### Auditory spatial localisation task

To assess participants’ accuracy in the spatial localisation of the tones, we calculated to what extent the spatial origin of the tone indicated by the participants deviated from its actual location, i.e., the Euclidean distance, in cm. This was calculated by means of the following formula (where X and Y indicate the coordinates on the horizontal and vertical dimension, respectively):

$$D=\sqrt{\left({\left(\left|X_{indicated\;origin}-X_{actual\;origin}\right|\right)}^{2}+{\left(\left|Y_{indicated\;origin}-Y_{actual\;origin}\right|\right)}^{2}\right)}$$

Thus, higher values indicate a poorer localisation accuracy, and lower values indicate a higher localisation accuracy. The mean deviation values were obtained by averaging the respective single deviation values, i.e., 16 values for each screen half for the analysis concerning the left and the right side, and 8 values for each vertical column for the subsequent, more fine-grained analysis.

To compare the mean absolute localisation deviation in the left half of the screen with the one in the right half of the screen, a paired-samples t-test was calculated. For the subsequent analysis on the vertical columns, a repeated-measures ANOVA was conducted on the mean absolute deviation values, with the within-subjects factor ‘column of the screen’ (levels: left peripheral, left central, right central, right peripheral). Moreover, to further analyse the direction of the deviation, we calculated relative mean deviation values, i.e., D-values were attributed a negative sign if the indicated origin of the tone was on the left of the actual origin (leftward deviation), and D-values were attributed a positive sign if the indicated origin of the tone was on the right of the actual origin (rightward deviation). Bonferroni-corrected one-sample t-tests were calculated to compare the mean relative deviation values against 0, and thus to investigate whether participants showed significant mean relative deviations when localising the origin of the tones. A repeated-measures ANOVA was then conducted on the mean relative deviation values, with the within-subjects factor ‘column of the screen’ (levels: left peripheral, left central, right central, right peripheral).

#### Search task with cross-modal spatial cueing

The search time (ST), i.e., the time elapsed between target presentation and the moment when participants touched the target, was recorded for each trial. For each participant, the mean STs were calculated for each target-tone spatial congruency condition, and each half of the screen (i.e., the STs for the 8 target locations within each half of the screen were averaged). In a first step, we conducted a repeated-measures ANOVA with the within-subjects factors ‘condition’ (levels: congruent, incongruent, mono sound, no sound) and ‘screen half’ (levels: left, right). Then, to further investigate the specific effects of various auditory cues on search performance, the no sound condition was considered as the baseline condition, and the mean ST of each of the other conditions (congruent, incongruent, mono sound) was expressed as a percentage of the search time for the respective screen side in the no sound condition. A resulting value of 100% would thus indicate equal STs in the no sound and the considered condition in the respective screen half, whereas values <100% indicate shorter STs in the considered condition, and values >100% indicate longer STs. Bonferroni-corrected one-sample t-tests were calculated to compare each of the resulting values for each of the three conditions (congruent, incongruent, mono sound) and each screen half against 100%, and thus to investigate whether there was a significant difference compared to the baseline (no sound) condition. A repeated-measures ANOVA with the within-subjects factors ‘condition’ (levels: congruent, incongruent, mono) and ‘screen half’ (levels: left, right) was then calculated. Subsequently, to further assess the obtained results, a more fine-grained analysis was conducted by dividing the touch-screen into four vertical columns (left peripheral, left central, right central, right peripheral) and expressing the results of each condition and column of the screen as a percentage of the ST for the respective column in the baseline (no sound) condition. Subsequently, Bonferroni-corrected one-sample t-tests were calculated to compare each of the resulting values for each of the three conditions (congruent, incongruent, mono sound) and for each column of the screen against 100%. A repeated-measures ANOVA with the within-subjects factors ‘condition’ (levels: congruent, incongruent, mono sound) and ‘column of the screen’ (levels: left peripheral, left central, right central, right peripheral) was then calculated.

It has previously been shown that reaction time data are typically non-normally distributed (e.g., [23, 24]). To address this issue, data transformations have been shown to reduce deviations from normality [25, 26]. In particular, the square root transformation has previously been successfully applied in reaction time studies [27, 28]. We thus transformed our search time data and our mean % change of search time data, respectively, by means of a square root transformation. All of the above mentioned analyses were then repeated on the transformed data. The analyses on square root transformed data showed the same patterns of results as the ones on untransformed data. In order to avoid redundancies and issues with the interpretation of transformed data [23], in the following we only report the results of the analyses on untransformed data.

#### Integration of tone localisation deviation into the search task with cross-modal spatial cueing

Pearson correlations were computed to assess whether participants’ tone localisation deviation was associated with their performance in the search task with cross-modal spatial cueing. More precisely, the absolute mean deviation values in each column of the screen during the auditory spatial localisation task were correlated with the mean STs in the same column during the congruent condition of the search task with cross-modal spatial cueing.

For the repeated-measures ANOVAs, all analyses, if the sphericity assumption was not met, the degrees of freedom (and thus the *p*-values) were corrected according to the Huynh-Feldt procedure. All post-hoc comparisons were performed by means of Bonferroni-corrected t-tests. Partial eta squared *η*_*p*_^2^ (for the repeated-measures ANOVAs), and Pearson’s correlation coefficient *r* (for post-hoc t-tests) were calculated as indicators of the effect sizes.

## Results

### Auditory spatial localisation task

**Fig 3:**
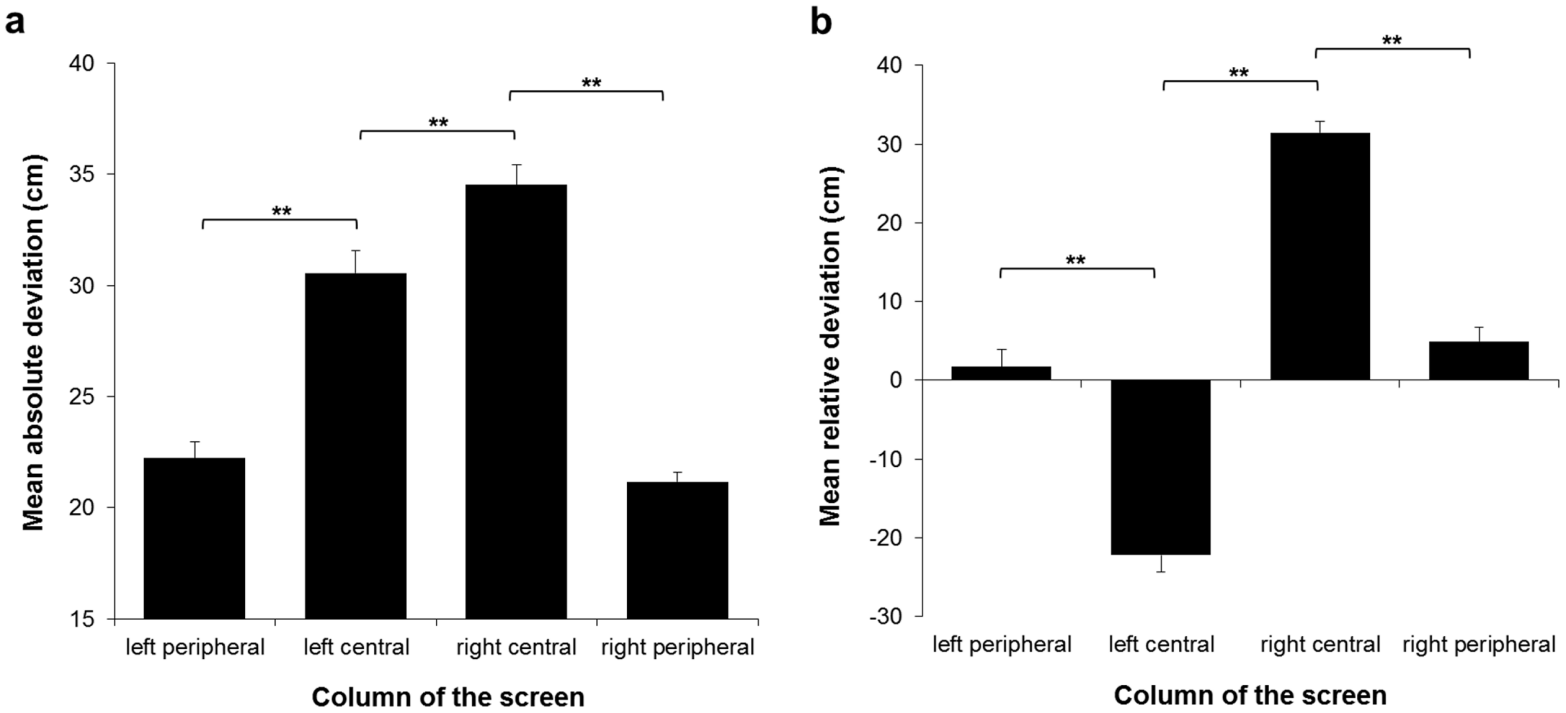
Auditory spatial localisation task. (a) Mean absolute deviation values, depicted as a function of the columns of the screen where tones were presented. Higher values indicate a higher mean localisation deviation and thus a lower mean localisation accuracy. (b) Mean relative deviation values, depicted as a function of the columns of the screen where tones were presented. Positive values indicate a rightward deviation, and negative values indicate a leftward deviation, when localising the origin of the tones. Asterisks represent significant post-hoc test (**p < .01; Bonferroni-corrected); only the most relevant post-hoc comparisons are depicted. Error bars represent the standard error of the mean (SEM).

The paired-samples t-test revealed a significantly lower mean absolute deviation for tones presented in the right compared to the left half of the touchscreen (t_56_ = -3.24, *p* = .002, *r* = .397). To further assess this result, we divided the touchscreen into 4 equal vertical columns. The repeated-measures ANOVA yielded a significant main effect of the factor ‘column of the screen’ (F_1.81,101.15_ = 71.49, *p* < .001, *η*_*p*_^2^ = .56). Post-hoc analyses revealed higher mean absolute deviation values for the left central and right central compared to the left peripheral and right peripheral columns, respectively (left central vs. left peripheral: t_168_ = 7.692, *p* < .001, *r* = .51; left central vs. right peripheral: t_168_ = 8.667, *p* < .001, *r* = .556; right central vs. left peripheral: t_168_ = 11.342, *p* < .001, *r* = .659; right central vs. right peripheral: t_168_ = 12.317, *p* < .001, *r* = .689). Moreover, the mean absolute deviation was higher in the right central compared to the left central column (t_168_ = 3.65, *p* = .002, *r* = .271). There was no significant difference in mean absolute deviation between the left and right peripheral columns (t_168_ = .975, *p* > .9; see Fig 3a). Concerning the mean relative deviation values, Bonferroni-corrected one sample t-tests revealed that the mean relative deviation significantly differed from 0 when localising tones presented in the left central (t_56_ = -10.304, *p* < .001, *r* = .809), right central (t_56_ = 21.976, *p* < .001, *r* = .947) and right peripheral columns (t_56_ = 2.725, *p* = .034, *r* = .342), yet not when presented in the left peripheral column (t_56_ = .794, *p* > .9). The repeated-measures ANOVA, which served to further investigate the direction of these deviations, also yielded a significant effect of the factor ‘column of the screen’ (F_1.52,85.1_ = 110.86; *p* < .001, *η*_*p*_^2^ = .66). Post-hoc tests are depicted in Fig 3b (right central vs. left central: t_168_ = 18.193, *p* < .001, *r* = .814; right central vs. left peripheral: t_168_ = 10.077, *p* < .001, *r* = .614; right central vs. right peripheral: t_168_ = 8.994, *p* < .001, *r* = .57; right peripheral vs. left peripheral: t_168_ = 1.083, *p* > .9; right peripheral vs. left central: t_168_ = 9.199, *p* < .001, *r* = .579; left peripheral vs. left central: t_168_ = 8.116, *p* < .001, *r* = .531). The mean relative deviation values demonstrated a rightward bias when localising tones originating from right central, and right peripheral columns, which was particularly pronounced for tones originating from the right central column. A leftward bias was observed for the localisation of tones originating from the left central column. However, no significant mean relative deviation was observed for the localisation of tones originating from the left peripheral column. Note, that tones originating from the central columns of the screen were localised less accurately than tones originating more peripherally, and that the pattern of localisation indicates a peripheral bias for the more centrally presented tones.

### Search task with cross-modal spatial cueing

**Fig 4:**
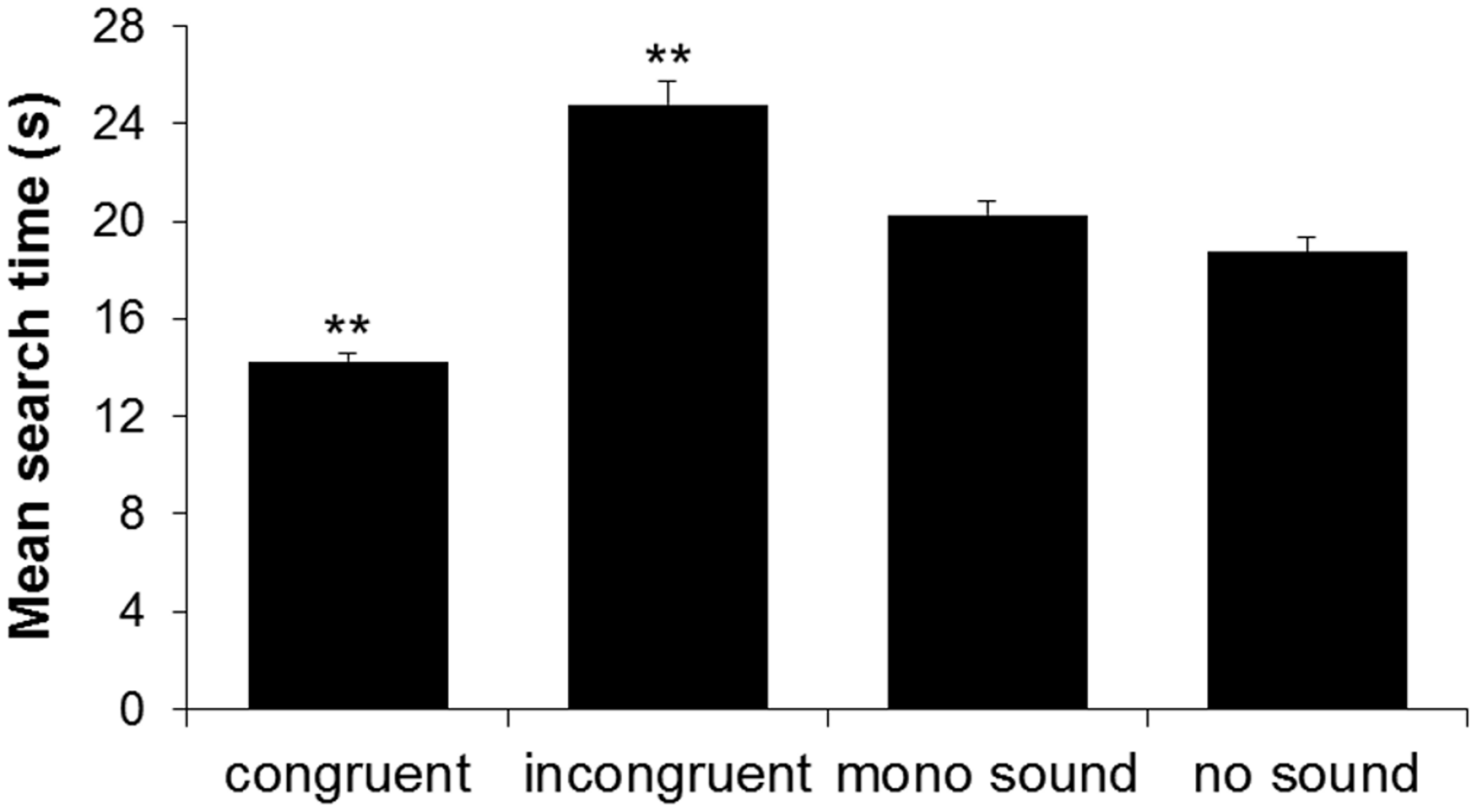
Mean search times in the search task with cross-modal spatial cueing. Mean search times (STs), depicted as a function of the condition of target-tone spatial congruency. Asterisks represent significant Bonferroni-corrected post-hoc tests (**p < .01). For the congruent and the incongruent conditions, asterisks indicate that all the Bonferroni-corrected post-hoc comparisons against the other conditions were significant. Error bars represent the SEM.

Concerning STs for visual targets presented in the left and the right halves of the touchscreen, the results revealed a significant main effect of the factor ‘condition’ (F_2.09,117.07_ = 79.17, *p* < .001, *η*_*p*_^2^ = .59) and a significant interaction between the factors ‘condition’ and ‘screen half’ (F_3,168_ = 6.57, *p* < .001, *η*_*p*_^2^ = .11), but no significant main effect of the factor ‘screen half’ (F_1,56_ = 2.06, *p* = .156). Regarding the main effect of the factor ‘condition’, the presentation of an auditory cue originating from the same spatial location as the target (i.e., the congruent condition) triggered a decrease in the mean STs, so that the search process under this condition of target-tone spatial congruency was significantly faster than in any other condition (congruent vs. incongruent: t_168_ = -10.779, *p* < .001; *r* = .806; congruent vs. mono sound: t_168_ = -6.153, *p* < .001, *r* = .429; congruent vs. no sound: t_168_ = -4.551, *p* < .001, *r* = .331). When the spatial locations of the auditory cue and of the visual target did not coincide (i.e., the incongruent condition), the mean STs were significantly longer than in any other condition (incongruent vs. mono sound: t_168_ = 4.626; *p* < .001, *r* = .336; incongruent vs. no sound: t_168_ = 6.228, *p* < .001, *r* = .433). Finally, when the visual search was preceded by a binaural tone with no spatial properties (i.e., the mono sound condition), mean STs did not significantly differ from the no sound condition (t_168_ = 1.602, *p* = .667; see Fig 4). Concerning the significant interaction between the factors ‘condition’ and ‘screen half’, post-hoc tests revealed a significant difference between the left and the right screen half only in the incongruent condition, i.e., visual targets presented in the right half were found significantly faster than visual targets presented in the left half of the touchscreen (t_168_ = -3.614, *p* = .016, *r* = .269). There were no significant asymmetries in the congruent (t_168_ = 0.4856, *p* > .9), mono sound (t_168_ = 1.869, *p* > .9), or no sound conditions (t_168_ = 2.302, *p* = .632).

**Fig 5:**
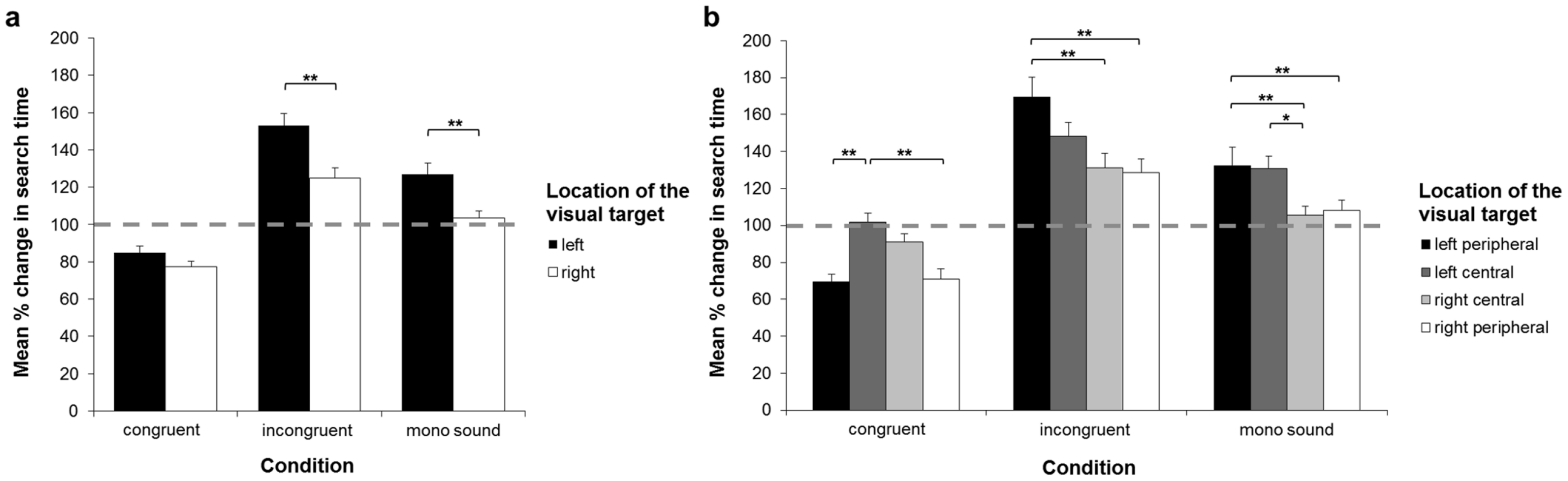
Mean % change in search times in the visual search task with cross-modal spatial cueing. Mean % change in search times (STs), calculated by expressing mean STs (a) of each half of the screen and (b) of each column of the screen of the congruent, incongruent and mono sound condition as a % of the ST in the no sound condition of the respective half or column of the screen. A value of 100% (indicated by the dashed, horizontal lines) represents equal STs as in the no sound condition. Asterisks represent significant Bonferroni-corrected post-hoc tests (*p < .05; **p < .01). Error bars represent the SEM. As indicated by Bonferroni-corrected one sample t-tests, compared to the no sound condition, there was a significant % change in STs for all of the conditions and target locations except for (a) targets presented in the right half of the screen in the mono sound condition, and (b) targets presented in the left and right central columns in the congruent condition and the right central and right peripheral columns in the mono sound condition.

For the subsequent analyses, the mean ST of each condition and screen half was expressed as a percentage of the mean ST of the no sound condition for the respective screen half. The one-sample t-tests revealed that the mean % change in STs for visual targets presented both in the left (congruent: t_56_ = -4.362, *p* < .001, *r* = .504; incongruent: t_56_ = 8.316, *p* > .001, *r* = .743) and in the right half (congruent: t_56_ = -7.713, *p* < .001, *r* = .718; incongruent: t_56_ = 4.485, *p* < .001, *r* = .514) of the screen significantly differed from 100% in the congruent and in the incongruent conditions, as did the mean % change in ST for targets presented in the left screen half in the mono sound condition (t_56_ = 4.339, *p* < .001, *r* = .502). The mean % change in ST for targets presented in the right half of the screen in the mono sound condition did not significantly differ from 100% (t_56_ = 0.972, *p* > .9). These results thus demonstrate significant differences compared to the baseline (i.e., the no sound condition) in all of the conditions and screen halves except for targets presented in the right half of the screen in the mono sound condition. The repeated-measures ANOVA yielded a significant main effect of the factors ‘condition’ (F_1.67,93.62_ = 102.33 *p* < .001, *η*_*p*_^2^ = .65) and ‘screen half’ (F_1,56_ = 12.16; *p* = .001, *η*_*p*_^2^ = .18) as well as a significant interaction between the two factors (‘condition*screen half’: F_2,112_ = 5.69, *p* = .004, *η*_*p*_^2^ = .09). Post-hoc tests revealed a higher mean % change in ST for targets presented in the left compared to the right half of the touchscreen, both in the incongruent (t_112_ = 6.145, *p* < .001, *r* = .502) and in the mono sound condition (t_112_ = 5.074, *p* < .001, *r* = .432). There was no significant asymmetry in the congruent condition (t_112_ = 1.586, *p* > .9; see Fig 5a). To further assess these asymmetries, we subdivided the touchscreen into 4 columns and expressed mean ST of each condition and column of the screen as a % of the ST in the no sound condition of the respective column. The one-sample t-tests revealed that, in the congruent condition, mean % change in STs in the left (t_56_ = .365, *p* > .9) and right central (t_56_ = -2.092, *p* > .491) columns did not significantly differ from 100%, nor did the mean % change in ST for the right central (t_56_ = 1.053, *p* > .9), and right peripheral (t_56_ = 1.397, *p* > .9) columns in the mono sound condition. All of the other mean % change in STs significantly differed from 100%, and thus from the baseline (i.e., the no sound) condition, whereby STs were decreased in the congruent, and increased in the incongruent and mono sound conditions (congruent, left peripheral: t_56_ = -7.463, *p* < .001, *r* = .499; congruent, right peripheral: t_56_ = -5.456, *p* < .001, *r* = .589; incongruent, left peripheral: t_56_ = 6.365, *p* < .001, *r* = .648; incongruent, left central: t_56_ = 6.524, *p* < .001, *r* = .657; incongruent, right central: t_56_ = 3.872, *p* = .003, *r* = .5, incongruent, right peripheral: t_56_ = 3.777, *p* = .005, *r* = .451; mono sound, left peripheral: t_56_ = 3.244, *p* = .023, *r* = .398; mono sound, left central: t_56_ = 4.607, *p* < .001, *r* = .524). The repeated-measures ANOVA yielded a significant main effect of the factors ‘condition’ (F_1.78,99.76_ = 102.03, *p* < .001, *η*_*p*_^2^ = .65) and ‘column of the screen’ (F_3,168_ = 4.55, *p* = .004, *η*_*p*_^2^ = .08), as well as a significant interaction of the two factors (‘condition*column of the screen’: F_5.45,305.17_ = 8.75, *p* < .001, *η*_*p*_^2^ = .14). Concerning this interaction, in the congruent condition, participants showed a significantly smaller mean % change in ST for visual targets presented in the left (t_336_ = -4.747, *p* < .001, *r* = .251) and the right (t_336_ = -4.522, *p* < .001, *r* = .24) peripheral columns compared to the left central column. In the incongruent condition, mean % change in ST for targets presented in the right central (t_336_ = -5.639, *p* < .001, *r* = .294) and right peripheral columns (t_336_ = -6.014, *p* < .001, *r* = .312) were significantly smaller compared to mean % change in ST for targets presented in the left peripheral column. Regarding the mono sound condition, post-hoc tests revealed significantly smaller mean % change in ST for visual targets presented in the right central (t_336_ = -3.958, *p* = .006, *r* = .211) and in the right peripheral (t_336_ = -3.577, *p* = .036, *r* = .019) column of the screen compared to the left peripheral column. Moreover, the mean % change in ST in the right central column was significantly smaller compared to the left central column (t_336_ = -3.727, *p* = .015, *r* = .199; see Fig 5b).

Taken together, the results of the search task with cross-modal spatial cueing revealed distinct patterns of spatial asymmetries according to the characteristics of the auditory cues. Crucially, when considering the no sound condition as a baseline, more fine-grained analyses revealed left/right asymmetries in the incongruent as well as in the mono sound condition, whereas such asymmetries were not detectable in the congruent condition.

### Integration of tone localisation deviation into the search task with cross-modal spatial cueing

**Fig 6:**
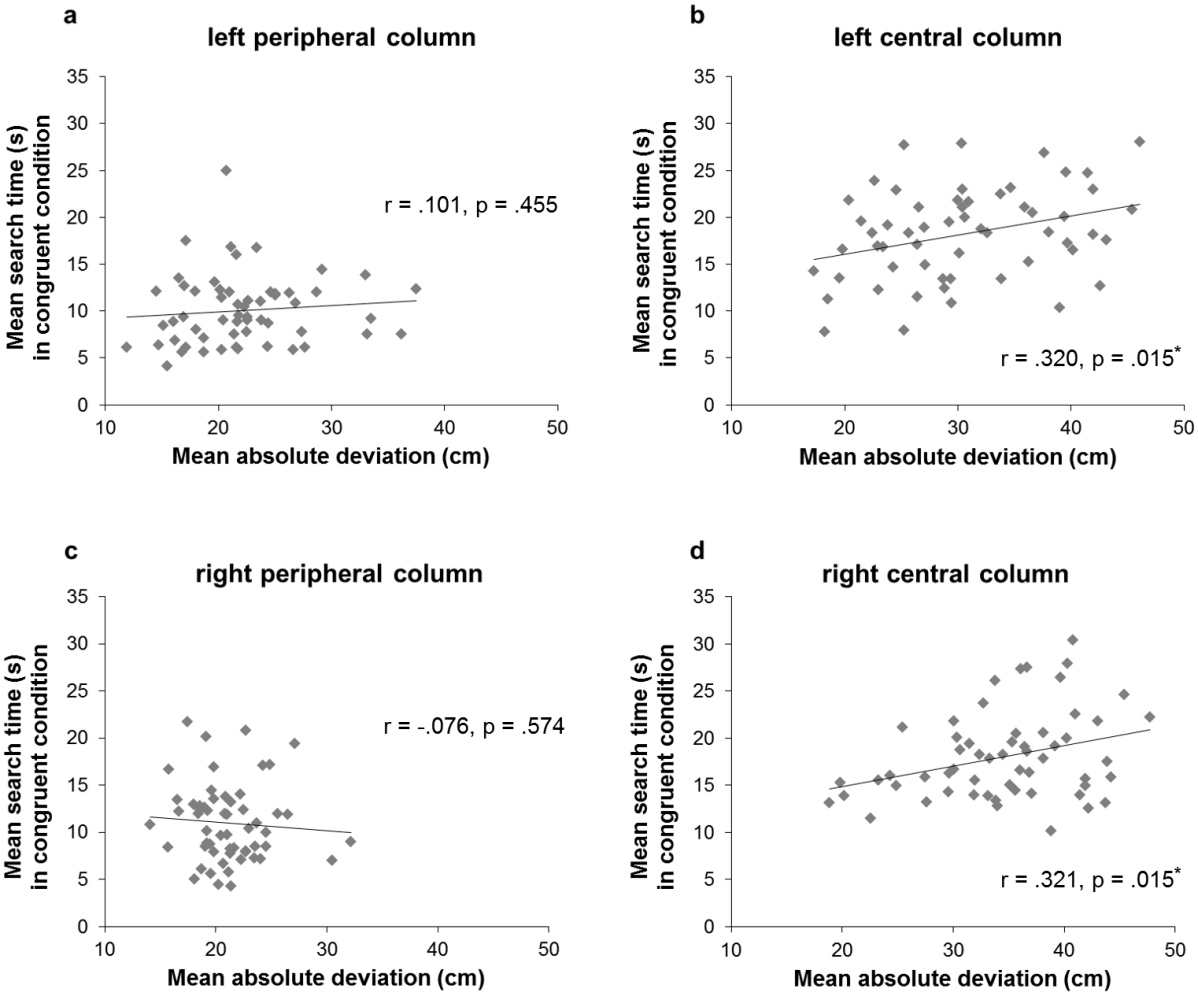
Correlations between localisation deviation and search times (STs) in the congruent condition. Correlations between the mean absolute deviation values (in cm) in the central and peripheral columns during the auditory spatial localisation task and STs in the same columns during the search task with cross-modal spatial cueing, in the congruent condition (i.e., tone originating from the same location of the target): (a) values for the left peripheral column; (b) values for the left central column; (c) values for the right peripheral column; (d) values for the right central column.

Regarding the relationship between participants’ tone localisation deviation and their mean STs in the congruent condition of the search task with cross-modal spatial cueing, significant correlations were found for the left and right central columns only. More precisely, in the congruent condition, there was a significant, positive correlation between the absolute mean deviation values when localising tones originating from the left central column and the mean STs in this same column (*r* = .320, *p* = .015). The same was true for the right central column (*r* = .321, *p* = .015). Hence, the more accurate participants were in localising tones in these central columns of the screen (i.e., the lower the deviation from the actual spatial origin), the faster they were at finding targets in the same columns in the congruent condition, i.e., when the targets were presented along with tones originating from the same position (see Fig 6).

No other correlations were significant, including the ones for the incongruent condition (search time for visual targets in the left peripheral column and auditory localisation deviation in the right central column: *r* = -.015, *p* = .914; search time for visual targets in the left central column and auditory localisation deviation in the right peripheral column: *r* = -.133, *p* = .323; search time for visual targets in the right central column and auditory localisation deviation in the left peripheral column: *r* = .145, *p* = .283; and, search time for visual targets in the right peripheral column and auditory localisation deviation in the left central column: *r* = -.055, *p* = .683).

## Discussion

The aim of the present study was to investigate whether spatial attentional asymmetries would occur in an audio-visual multisensory setting, namely in an overt search task with cross-modal spatial cueing. In the unimodal, auditory spatial localisation task, i.e., when localising the origin of a tone without the presence of visual stimuli, participants demonstrated higher accuracies for tones originating from peripheral (left and right) areas compared to central (left and right) areas. Moreover, tone localisation was more accurate in the left central compared to the right central area. In the unimodal visual (i.e., no sound) condition of the search task, participants demonstrated no left/right asymmetries in search times. Yet, crucially, the additional presentation of an incongruent auditory cue, as well as of a spatially non-informative auditory cue, resulted in significant lateral asymmetries. Thereby, STs were prolonged for visual targets located in the left compared to the right area of the search field. In contrast, such asymmetries were not present in the congruent condition. The additional presentation of a spatially congruent auditory cue triggered equally decreased STs for visual targets presented in the left and the right peripheral areas of the search array. Moreover, significant correlations were found between STs for visual targets in the central areas of the search array and tone localisation accuracy within these same areas.

In the following, we will first discuss the results of the unimodal auditory spatial localisation task, then focus on the more general findings of the search task with cross-modal spatial cueing, and finally discuss the novel findings of the observed spatial asymmetries in the search task with cross-modal spatial cueing.

### Auditory spatial localisation

The completion of the auditory spatial localisation task enabled the investigation of participants’ localisation accuracy per se, i.e., without a competing visual task. In turn, this also served as an assessment of how localisation accuracy could influence auditory cueing efficacy. Overall, the results demonstrated a higher localisation accuracy for tones originating from the left and the right periphery, as compared to the left and the right central areas. Indeed, tones originating from the left and the right central areas of the screen were localised more peripherally than their actual spatial origin, thus nicely demonstrating the peripheral bias previously observed in several sound localisation tasks [22, 29–32]. The absence of this peripheral bias for the left and the right peripheral columns is attributable to the physical limits of the screen, making a more peripheral localisation of the presented tones impossible. Interestingly, the peripheral bias observed for the central areas was more pronounced in the right compared to the left central column, i.e., participants localised sounds more accurately in the left compared to the right central column. A possible explanation for this finding can be derived from previous studies (e.g., [33, 30]) that have found a slight leftward bias in auditory space perception, particularly in right-handers. This bias has been associated with the right-hemispheric dominance for visuo-spatial attention ([33], see also [34], [35]). Hence, in our study, participants might have shown a slight leftward bias in auditory spatial attention, leading to a more precise localisation of sounds presented in the left central compared to the right central column.

### Main effect of target-tone congruency in the audio-visual search task

In the unimodal visual (i.e., no sound) condition of the search task, the results confirmed the high perceptual demands of the task, as participants demonstrated long STs (on average, approximately 18.7 seconds per trial). The congruent cross-modal cueing condition, i.e., when the origin of the auditory cue and the location of the visual target coincided, resulted overall in the fastest STs. Such an auditory spatial facilitation has frequently been reported (e.g., [3–5, 7, 36–39]), and can be explained by means of an attentional shift to the perceived origin of the sound, thereby enhancing visual attentional processing, and hence visual target detection, in the respective area [9, 36]. The incongruence between the spatial origin of the auditory cue and the visual target resulted in overall significantly longer STs. Such an impairing effect of invalid auditory cues on visual target detection is compatible with numerous previous lines of research (e.g., [2, 15, 8, 36]). This effect has been explained in terms of the allocation of increased attentional resources, and enhanced processing, at the incorrectly (i.e., non-target) cued location, due to an attentional shift to this location. Attention has thus to be disengaged from the incorrect location and shifted (i.e., reoriented) to the target location, resulting in increased reaction times [40]. Interestingly, the presentation of a tone with no spatial information (i.e., a mono sound) triggered no significant differences in STs compared to the no sound condition. Similar results were reported by Dufour [2], who did not find a difference in target detection accuracy when comparing the impact of spatially non-informative sounds with a no sound condition. Some studies have proposed that the presentation of a non-spatial auditory stimulus can act as a warning, and temporarily increase alertness, which would in turn lead to shorter reaction times in target detection tasks [41, 42]. However, such an alerting effect has been shown to last only for short time intervals (i.e., several hundreds of ms [43]), and tasks investigating potential alerting effects typically involve simpler target detection paradigms (e.g., [42, 44]). Our task was more complex and, moreover, the frequent presentation of tones (i.e., in all of the conditions, except for the no sound condition) probably rendered any potential alerting effect of the tone ineffective. Accordingly, in the present study, the effects of spatial auditory cues on participants’ visual search performance are unlikely to be due to an alerting effect.

### Spatial asymmetries in the audio-visual search task

The aim of the present study was to investigate whether spatial asymmetries occur in an audio-visual search task. To the best of our knowledge, this aspect has not yet been examined by previous studies. The results of the search task with cross-modal spatial cueing, according to the hemifield in which the visual target was presented, revealed no left/right asymmetries in the unimodal visual (no sound) condition. When considering this condition as the baseline, which served to compare the specific effects of different types of auditory cues, the addition of an incongruent or of a spatially non-informative auditory cue to the search task resulted in significant lateral asymmetries. Thereby, STs were longer for visual targets presented in the left compared to the right half of the search array. More detailed analyses of the mono sound condition revealed that this asymmetry was due to an increase in STs in both the left central and left peripheral areas of the search field, whereas there were no significant changes for targets presented in the right half of the search field. In the incongruent condition, though STs for targets presented in all of the areas of the search field increased, this increase was largest for targets presented in the left periphery, ultimately resulting in the observed left/right asymmetry. The results suggest that these spatial asymmetries in the STs are due to an increase in the costs associated with the detection of targets presented in the left hemifield, rather than to a decrease in STs for targets presented in the right hemifield.

A possible interpretation for the observed left/right asymmetries in the incongruent, as well as in the mono sound, conditions is based on an attentional bias, favouring the deployment of attention into the right over the left visual space. In line with this explanation, left/right asymmetries have also been found in a visuo-tactile cueing paradigm [18], with a larger difference between invalidly and validly cued responses for the left compared to the right hemifield. In fact, based on findings of multisensory studies with visuo-tactile and audio-visual processing tasks ([18, 19, 45, 17], data reanalysed in [16]), it has recently been proposed that attentional asymmetries might occur in multisensory settings with high perceptual load, and when the tasks require multisensory processing at later stages (and is thus more prone to top-down attentional modulation). The results of the present study further imply that such asymmetries might particularly occur when attention has to be reoriented. In the incongruent condition, attention had to be reoriented towards the opposite hemifield after the presentation of the auditory cue. Slower reaction times for leftward attentional reorienting have also been found in a unimodal visual cueing task [46]. Moreover, previous research has demonstrated that reaction times become longer with an increasing spatial distance between an invalid auditory cue and a visual target, both within and across hemifields ([47, 48], see also [49] for differences within- and between hemifields). Thus, the results observed in the mono sound condition could potentially be explained by an attempt of the participants to localise the origin of the sound. The mono sound would most likely be localised in the centre of the screen, due to the equal amount of auditory information presented to the left and the right ear. To somehow substantiate this hypothesis, we asked an independent, small group of participant to localise the spatial origin of the same mono sound used in our experiment. These participants indeed localised the mono sound near to the centre of the touchscreen (N = 6; M = 952.9 pixels, SEM = 39.53; horizontal middle of the screen = 960 pixels). The smaller costs of reorienting observed in the mono sound compared to the incongruent condition could thus be explained by means of the different distances between the localisation of the auditory cue and the visual target, i.e., assumedly central in the mono sound condition, and in the opposite hemispace in the incongruent condition.

Thus, the results of the present study support the aforementioned proposal that multisensory processing can lead to the occurrence of a rightward attentional bias. Yet, the origin of this bias is still unclear, and has been proposed to be due to attention *per se* being rightward-biased, or to *cross-modal* attention being rightward-biased [16, 19]. The results of our study, including both unimodal and cross-modal search conditions, seem not to support either of these explanations. Concerning the former (i.e., attention *per se* being rightward-biased), in this case one would expect to observe an attentional asymmetry in the unimodal, visual search condition without any auditory cues. Yet, such an asymmetry was absent in our results. Concerning the latter (i.e., *cross-modal* attention being rightward-biased), in this case one would also expect to observe left/right asymmetries in the congruent cross-modal condition. Again, such an asymmetry was absent in our results. Thus, our findings suggest that spatial attentional biases in multisensory processing are not generalised, but seem to arise under specific attentional conditions, namely when attention has to be reoriented. In order to further substantiate this hypothesis, future studies should directly compare the effects of unimodal spatial cueing vs. cross-modal spatial cueing on search performance.

Interestingly, the results revealed no left/right asymmetries in the congruent condition. In fact, detailed analyses, considering the no sound condition as the baseline, demonstrated that participants particularly benefitted from the presentation of a congruent auditory cue when visual targets were localised in the left or right peripheral areas of the visual search field, the same areas for which they showed higher tone localisation accuracies. A larger benefit of congruent tones on target detection performance with increasing target eccentricity is also compatible with the results of previous studies (e.g., [3, 50]). Moreover, on a group level, the participants did not significantly benefit from congruent auditory cues when the visual targets were presented in the left or the right central areas of the visual search field, the same areas in which their auditory localisation capability was considerably less accurate. However, the observed significant correlations between auditory localisation accuracy and STs in the central search field (i.e., the higher the localisation accuracy, the shorter the STs), further stress the importance of an accurate localisation capability in order to benefit from audio-visual congruency in complex search tasks with cross-modal spatial cueing.

In the present study, due to the ratio of the presentation of congruent, incongruent, and mono sounds (i.e., 3-1-2), the auditory cues were mostly informative, and attention was thus cued endogenously. However, several previous studies applied cross-modal exogenous attentional cueing (i.e. when the auditory cue is not predictive of the location in which the visual target is probable to occur), in which attention is captured “automatically” by an auditory cue [15]. These studies typically found that congruent auditory cues have beneficial effects, whereas incongruent auditory cues (occurring from the opposite hemifield) have detrimental effects on visual target detection performance (e.g., [2, 35, 51]). These results are similar to the ones of our study and of other previous studies using cross-modal endogenous cueing paradigms (e.g. [32, 49]). It seems thus reasonable to hypothesize that the spatial attentional asymmetries observed in our study with endogenous cross-modal cueing may also be present when applying exogenous cross-modal cueing. This hypothesis should, however, be specifically tested in future studies.

### Practical implications

Overall, the results of the present study may also have practical implications. The finding that spatially congruent auditory cues facilitate the detection of visual targets has led researchers to propose their implementation in everyday tasks that pose high demands on the visual modality (e.g., driving [52]). Moreover, the results of the present study might also be of relevance for patients with attentional disorders, such as patients with visual hemispatial neglect. Patients with visual neglect show impaired target detection in the visual field contralateral to the lesioned hemisphere, which is exacerbated by increasing attentional load (e.g., [53, 54]). Thus, the potential beneficial effects of congruent auditory cues on neglect patients’ visual attentional deployment could aid in the improvement of neurorehabilitative methods (see also [55]). Thereby, one should also be aware that incongruent or spatially non-informative auditory cues could potentially increase attentional asymmetries in neglect patients, particularly in patients with left-side neglect, since, as shown in the present study, such cues can elicit left/right differences already in healthy individuals.

## Conclusion

In conclusion, the findings of the present study demonstrate that multisensory processing, i.e., cross-modal spatial cueing in a visual search task, can result in spatial asymmetries, depending on the validity of the cross-modal cue. A left/right asymmetry emerged when the origin of the auditory cue and the location of the visual target were spatially incongruent, and when the auditory cue contained no spatial information. This left/right asymmetry was due to costs associated with the reorienting of attention towards the left hemifield. No left/right differences, but rather an equal benefit for the left and the right peripheral search areas, were observed when the origin of the auditory cue and the location of the visual target coincided. Moreover, search performance in the congruent condition was associated with the localisation accuracy of the auditory cues. These findings are of potential relevance for healthy individuals’ performance in everyday tasks that pose high demands on the visual modality, and for clinical populations with attentional disorders, such as hemispatial neglect patients.

## Supporting information

S1 Dataset Data of the cross-modal search task and the auditory spatial localisation task. (XLSX)
